# Effect of Diffusion on the Ultimate Axial Load of Complex-Shaped Al-SiC Samples: A Molecular Dynamics Study

**DOI:** 10.3390/molecules29143343

**Published:** 2024-07-16

**Authors:** Mostafa Fathalian, Eligiusz Postek, Masoud Tahani, Tomasz Sadowski

**Affiliations:** 1 Institute of Fundamental Technological Research, Polish Academy of Sciences, Pawinskiego 5B, 02-106 Warsaw, Poland; epostek@ippt.pan.pl (E.P.); mtahani@um.ac.ir (M.T.); 2 Department of Mechanical Engineering, Ferdowsi University of Mashhad, Mashhad 9177948974, Iran; 3 Department of Solid Mechanics, Lublin University of Technology, 20-618 Lublin, Poland

**Keywords:** molecular dynamic, Al-SiC composites, diffusion, SiC particle

## Abstract

Metal matrix composites (MMCs) combine metal with ceramic reinforcement, offering high strength, stiffness, corrosion resistance, and low weight for diverse applications. Al-SiC, a common MMC, consists of an aluminum matrix reinforced with silicon carbide, making it ideal for the aerospace and automotive industries. In this work, molecular dynamics simulations are performed to investigate the mechanical properties of the complex-shaped models of Al-SiC. Three different volume fractions of SiC particles, precisely 10%, 15%, and 25%, are investigated in a composite under uniaxial tensile loading. The tensile behavior of Al-SiC composites is evaluated under two loading directions, considering both cases with and without diffusion effects. The results show that diffusion increases the ultimate tensile strength of the Al-SiC composite, particularly for the 15% SiC volume fraction. Regarding the shape of the SiC particles considered in this research, the strength of the composite varies in different directions. Specifically, the ultimate strength of the Al-SiC composite with 25% SiC reached 11.29 GPa in one direction, and 6.63 GPa in another, demonstrating the material’s anisotropic mechanical behavior when diffusion effects are considered. Young’s modulus shows negligible change in the presence of diffusion. Furthermore, diffusion improves toughness in Al-SiC composites, resulting in higher values compared to those without diffusion, as evidenced by the 25% SiC volume fraction composite (2.086 GPa) versus 15% (0.863 GPa) and 10% (1.296 GPa) SiC volume fractions.

## 1. Introduction

In the past two decades, metal matrix composites (MMCs) have evolved from a topic of scientific interest to a material of significant technological and commercial importance, coinciding with publications in the domain of composite science and technology. MMC consists of metals reinforced by additional metals, ceramics, or organic compounds. As the demand for lightweight materials rises in aviation, aerospace, transportation, and electronics industries, MMCs have gained significant traction due to their outstanding mechanical properties [1,2]. Because of their fundamental and versatile properties, aluminum metal matrix composites (AMCs) are well-suited for manufacturing various components, including engine parts, due to their lightweight nature [3,4,5,6]. Among MMCs, AMCs reinforced with ceramic particles like SiC have emerged as a preferred choice in various industrial sectors. This preference is attributed to their high specific modulus, exceptional strength, excellent wear resistance, and superior thermal conductivity [7,8,9].

Despite numerous experiments, there still exists an incomplete understanding of the microstructural alterations occurring in the SiC particle-reinforced composites [10]. This partial understanding is primarily attributed to the challenges of detecting these changes in situ using laboratory equipment. To gain a deeper insight into the mechanical properties of Al-SiC composites, various simulation techniques have been utilized to investigate the behavior of the composites reinforced with SiC particles [10,11,12]. Among all the atomistic simulation methods, molecular dynamics (MD) simulation is very effective in modeling composites for mechanical properties [13,14]. Out of the numerical codes developed for atomistic simulations, LAMMPS (30 July 2021 version) [15,16] stands out as the open-source software that has garnered significant interest in the scientific community due to its exceptional flexibility, wide range of functionalities, and responsive community support.

Several MD simulation investigations have been conducted on aluminum matrix composites (Al-SiC) reinforced with SiC particles. For example, Huo et al. [17] conducted research on the atomistic scale to investigate the mechanical properties and strengthening mechanisms of Al/SiC composites. The result shows that the dispersion of SiC particles within the Al matrix causes a significant increase in the strength of composites compared to pure Al. Liu et al. [18] investigated the residual stress of the Al-SiC interface. As a result of nanoindentation, there is a large microscopic residual stress concentration at the indentation area. The microscopic residual stress on the SiC side in the indentation region is more serious than that on the Al side. Vu et al. [19] examined the deformation behavior of aluminum polycrystalline reinforced with SiC particles. The study demonstrated that the grain boundary and Al-SiC interface play pivotal roles in the mechanical characteristics of the composite. Higher strain rates lead to the increased formation of hexagonal close-packed and amorphous structures, greater dislocation density, and material failure. Lin et al. [20] evaluated the dynamic strength, reinforcing mechanism, and damage characteristics of Al-SiC composites by MD simulation. They investigated the behavior of dislocation motion and interaction under the shock loading of SiC-Al composites. The result showed that dynamic compressive strength depends on changes in pressure and the development of dislocation structures. Tahani et al. [21] investigated the influence of diffusion at the interface of Al-SiC composites. While this research focused solely on simple interfaces, the results revealed that diffusion enhances the strength at the interface of Al-SiC composites. 

Prior research has demonstrated that diffusion leads to the forming of an aluminum–silicon carbide phase layer at the interface, impacting the strength of both the interface and the composite. This research reveals the role of diffusion in strengthening Al-SiC composite interfaces with complex shapes, a factor neglected in previous studies focused solely on simple interfaces. In this work, a main SiC column was modeled with eight connected sub-columns of SiC. Then, the SiC structure was merged with Al to make the Al-SiC composite. Three different volume fractions of SiC consisting of 10%, 15%, and 25% were considered. Tensile deformation was simulated in two orthogonal directions (likely x and z) to evaluate the composites’ mechanical behavior. Young’s modulus, ultimate strength, and toughness were analyzed, considering both scenarios with and without diffusion effects.

## 2. Simulation Results and Discussion

### 2.1. Effects of Diffusion on the Young’s Modulus of Al-SiC Composite

**Figure 1:**
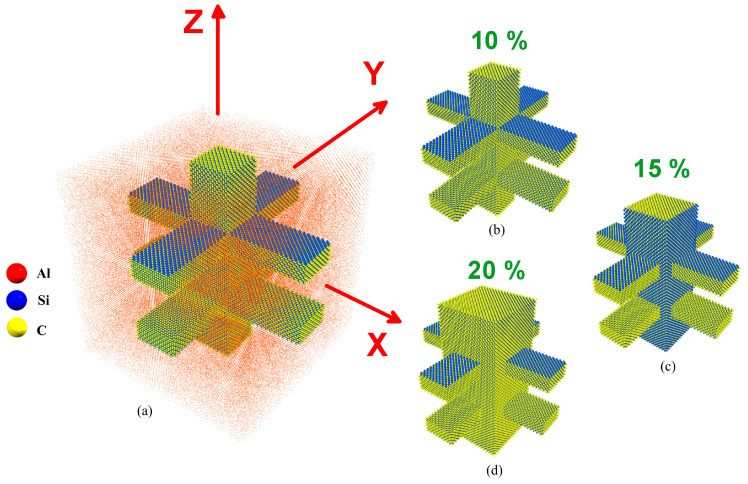
(**a**) Perspective view of an Al-SiC composite, (**b**) SiC structure with 10% volume fraction, (**c**) SiC structure with 15% volume fraction, and (**d**) SiC structure with 25% volume fraction.

Models based on Al-SiC composites with varying SiC volume fractions of 10%, 15%, and 25% (as shown in Figure 1) are investigated with and without considering diffusion. 

The potential energy is determined using the MD method.

**Figure 2:**
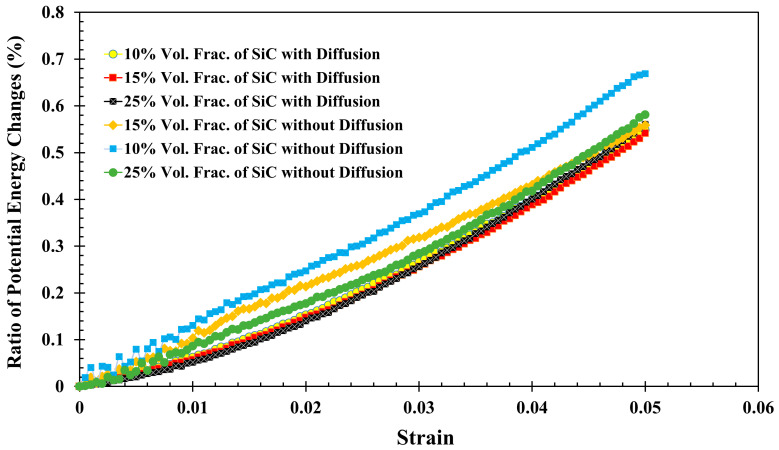
Potential energy versus strain for the uniaxial strain of an Al-SiC composite at three volume fractions of SiC: 10%, 15%, and 25%, including both with and without the presence of diffusion.

Figure 2 presents potential energy versus strain for the uniaxial strain of an Al-SiC composite at three volume fractions of SiC: 10%, 15%, and 25%, including both with and without the presence of diffusion. The resulting dataset was then fitted to a second-order polynomial equation to accurately describe the potential energy and strain relationship. The quadratic nature of the polynomial allows for the precise characterization of the system’s response to deformation, and the coefficients of this polynomial were subsequently used to calculate Young’s modulus. In this method, the second-order polynomial equation is *U*(*ε*) = *aε*^2^ *+ bε + c*, where *U*(*ε*) is the potential energy, ε is the strain, and *a*, *b*, and *c* are the polynomial coefficients determined from the curve fitting. Young’s modulus can then be calculated using the second derivative of the potential energy with respect to strain, evaluated over the supercell volume [22,23,24,25,26],

(1) $$E=\frac{1}{V}\left(\frac{d^{2}U}{d\varepsilon^{2}}\right)$$

where *V* represents the volume of the Al-SiC supercell, *U* denotes the potential energy, and *ε* is defined as ∆*L/L*_0_, where ∆*L* is the change in bulk length relative to the initial bulk length.

Figure 2 represents the percentage change in potential energy versus strain. This approach allows for a more effective analysis of relative changes in potential energy. Specifically, the original potential energy values (in eV) are converted to percentage growth curves. The reference point is the initial potential energy at the curve’s starting point. The reference points for Al-SiC considering diffusion are −1,644,250 eV, −1,776,629 eV, and −1,966,135 eV for 10%, 15%, and 25% volume fractions of SiC, respectively. Additionally, the reference points for Al-SiC without the consideration of diffusion are −1,597,680 eV, −1,720,729 eV, and −1,915,338 eV for the same volume fractions of SiC. For each subsequent point on the curve, the current value represents the potential energy at that point. The percentage growth for each point is calculated using the following formula:

(2) $$Percentage\;Growth=\left(\frac{Current\;Value-Reference\;point}{Reference\;point}\right)\times 100$$

Table 1 shows the effect of diffusion on Young’s modulus for an Al-based composite with 10%, 15%, and 25% of SiC as a reinforcement. As a result of the diffusion effect, for the Al-SiC composite, Young’s moduli of 123.72 GPa, 135.21 GPa, and 141.32 GPa correspond to the volume fractions of 10%, 15%, and 25% of SiC, respectively. Moreover, Young’s modulus for the Al-SiC composite is 120.42 GPa, 132.32 GPa, and 139.11 GPa for 10%, 15%, and 25% SiC, respectively, regardless of diffusion. According to the following table’s results, diffusion has a negligible effect on Young’s modulus for all the volume fractions of particles. Although diffusion between the aluminum matrix and SiC particles can potentially enhance bonding at the interface, the results indicate that this reinforced bonding does not significantly increase stiffness. 

### 2.2. Tensile Test Simulation

Aiming at the investigation of the influence of SiC volume fraction and diffusion effects on the deformation mechanism of the Al matrix, the ultimate strength of the Al-SiC composite with different SiC volume fractions (10%, 15%, and 25%) was compared, both with and without considering the diffusion effect. Since the Al-SiC composite models in this study have different shapes in the x and z directions, the tensile test was conducted in two different directions (x and z).

**Figure 3:**
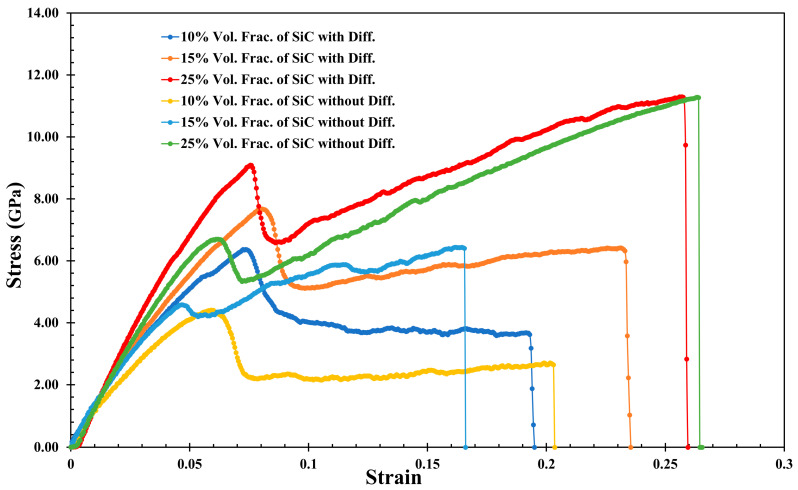
Tensile stress–strain curves for the Al-SiC composite with three SiC volume fractions (10%, 15%, and 25%) considering and neglecting the diffusion effect in the z-direction.

Figure 3 displays the stress–strain relationship for the various volumes of SiC in the Al-SiC composite, both with and without diffusion, in the z-direction. Additionally, Table 2 presents the results for the different volume fractions of SiC in the Al-SiC composite, again with and without diffusion in the z-direction. With the presence of diffusion, the highest ultimate strength is related to the Al-SiC composite containing a 25% volume fraction of SiC (11.29 GPa). In comparison, the lowest ultimate strength is of the Al-SiC composite containing a 10% volume fraction of SiC (5.93 GPa). The combination of higher reinforcement content, improved interfacial bonding, potential diffusion effects, and microstructural differences likely contributes to the higher ultimate strength observed in the Al-SiC composite with a 25% volume fraction of SiC compared to the composite with a 10% volume fraction of SiC. The Al-SiC composite with a 25% volume fraction of SiC exhibits higher toughness (2.086 GPa) compared to the composites with a 15% volume fraction of SiC (0.863 GPa) and a 10% volume fraction of SiC (1.296 GPa), respectively. While the general trend suggests that increasing SiC content in the composites leads to the increased fracture toughness, the complex shape of Al-SiC composites can influence toughness through various mechanisms related to stress concentration, load distribution, boundary effects, material distribution, and geometry-dependent toughening mechanisms.

When diffusion is absent, strength significantly decreases, particularly in the Al-SiC composites with 10% and 15% volume fractions of SiC, compared to when diffusion is considered (4.105 GPa and 6.110 GPa, respectively). The presence of diffusion processes plays a pivotal role in improving the strength of the Al-SiC composites by promoting better interfacial bonding, reducing stress concentrations, optimizing the microstructure, and inducing strengthening mechanisms. In contrast, when diffusion is absent, these beneficial effects are diminished or absent, resulting in decreased strength, particularly in the composites containing lower SiC content, such as those with 10% and 15% volume fractions of SiC. Without considering the diffusion effect, the curves generally indicate a decrease in toughness. While SiC reinforcement can strengthen Al-SiC composites, neglecting diffusion effects can lead to a decrease in toughness. When the diffusion is absent, the bond between the SiC and the aluminum is weak. It allows the high stress to concentrate more intensely around the particles, further promoting crack initiation. In other words, without diffusion to create a robust interface, these stresses cannot be effectively distributed, resulting in early crack initiation and brittle failure. The trend in these results aligns with the findings of previous studies [21,27].

The stress–strain curve for the Al-SiC composite with a 25% volume fraction of SiC and diffusion (Figure 3) exhibits two distinct peaks before reaching the failure point. Analyzing the corresponding microstructural evolution depicted in Figure 3 is essential to understanding the potential mechanisms behind these peaks. 

**Figure 4:**
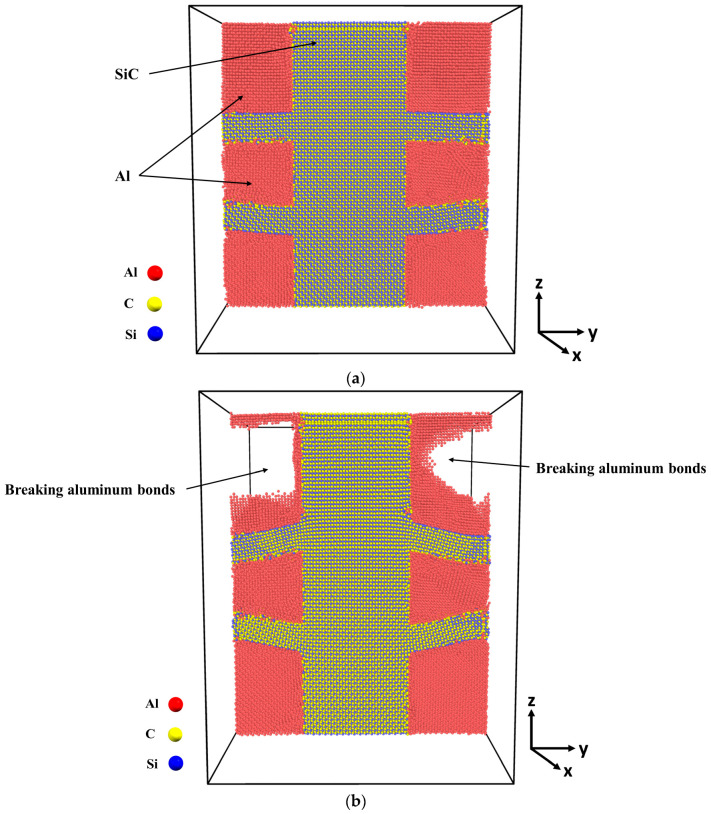

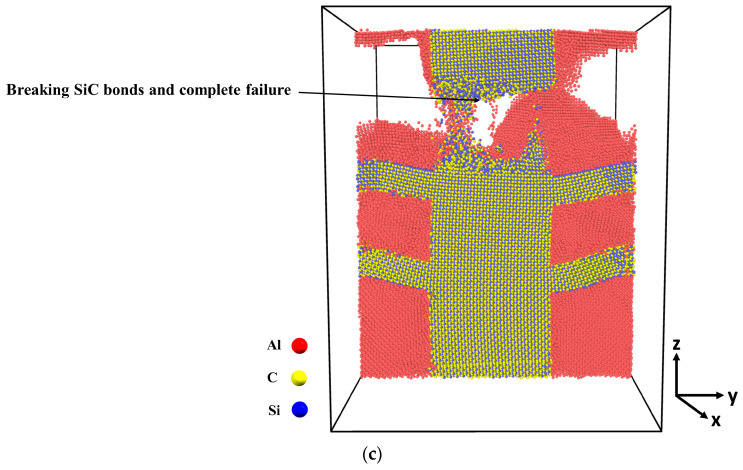
Deformed cross-section of the Al-SiC composite with a 25% volume fraction of SiC under tensile deformation ($\sigma_{zz})$ in the z-direction $\left(\varepsilon_{zz}\right)$ with diffusion: (**a**) Microstructure at the first peak of the stress–strain curve (Figure 3). (**b**) Microstructure at the second peak of the stress–strain curve (Figure 3). (**c**) Microstructure after complete failure.

Figure 4 presents the microstructure of the Al-SiC composite with a 25% volume fraction of SiC and diffusion at different stages of tensile deformation, directly corresponding to the two stress peaks observed in Figure 3. The stress–strain curve (Figure 3) shows a first peak at 9 GPa, followed by a significant decrease in stress. Figure 4a corresponds to the initial peak of the stress–strain curve for the Al-SiC composite with a 25% volume fraction of SiC and diffusion. It reveals no signs of debonding at the interface between the Al and SiC sides. Then, starting from a strain of 0.09, the stress gradually increases until it reaches a strain of 0.257 (stress is 11.2 GPa), as depicted in Figure 3. Figure 4b corresponds to the second peak and reveals debonding only on the Al side. Figure 4c illustrates the complete failure of the Al-SiC composite under uniaxial tensile load. This failure is characterized by debonding on the SiC side.

In the stress–strain curve of the Al-SiC composite, the presence of two peaks could be attributed to the unique geometric characteristics of the composite material. The appearance of the first peak may be influenced by the specific geometry of the composite, where initial failure occurs primarily in the Al region due to certain stress concentrations or material properties. Subsequently, the second peak may reflect the response of the remaining SiC structure, which continues to resist tensile loading after the initial failure in the Al phase.

**Figure 5:**
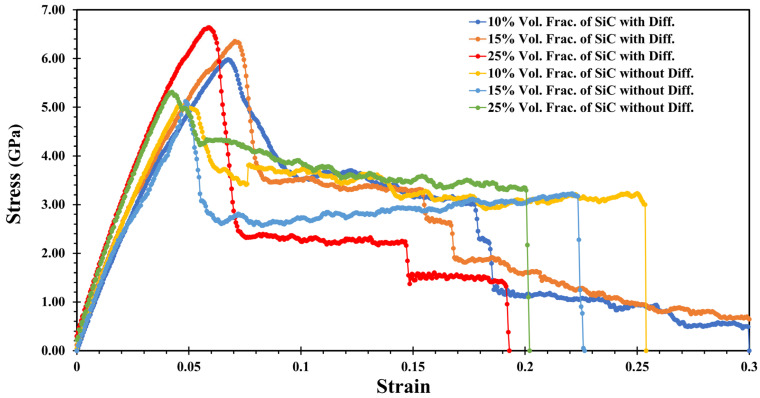
Tensile stress–strain curves for the Al-SiC composite with three SiC volume fractions (10%, 15%, and 25%) considering and neglecting the diffusion effect in the x-direction.

Figure 5 shows tensile stress–strain curves for three different volume fractions of SiC (10%, 15%, and 25%) with and without diffusion under x-direction tensile loading. Table 3 presents the results of ultimate strength and toughness for the Al-SiC composites with varying SiC volume fractions, focusing on the influence of diffusion in the x-direction. In the diffusion section, the highest strength is observed in the Al-SiC composite with a 25% volume fraction of SiC (6.63 GPa). The second highest ultimate strength is observed for the Al-SiC composite with a 15% volume fraction of SiC at 6.35 GPa. Among the Al-SiC composites, the composite with a 10% volume fraction of SiC shows the lowest ultimate strength (5.98 GPa). The toughness of the 10% and 15% volume fractions has not changed dramatically, but the toughness of the 25% volume fraction has decreased significantly. The decrease in toughness observed at a 25% SiC volume fraction can be attributed to increased stress concentrations, reduced stress transfer efficiency (the transmission of mechanical stress between the Al and SiC), and the transition towards a more brittle behavior due to the higher proportion of the SiC particles.

The maximum ultimate strength for stress–strain curves without diffusion is observed for Al-SiC with a 25% volume fraction at 5.31 GPa, compared to 5.12 GPa with a 15% volume fraction. As the volume fraction of SiC increases, there is a significant increase in ultimate strength, as illustrated in Figure 4 and Table 3. Toughness for Al-SiC with 10% SiC content is 0.857 GPa, for Al-SiC with 15% SiC content is 0.673 GPa, and 0.788 GPa for 25% SiC content.

### 2.3. Analyzing Local Stress Distribution in Al-SiC Composites under Tensile Deformation

This section aims to better understand how diffusion affects local stress in the Al-SiC interfaces during tensile deformation. Two snapshots are considered: stress per atom without diffusion, and stress per atom considering diffusion. For this purpose, composites with a 10% volume fraction of SiC, both with and without diffusion, are employed.

**Figure 6:**
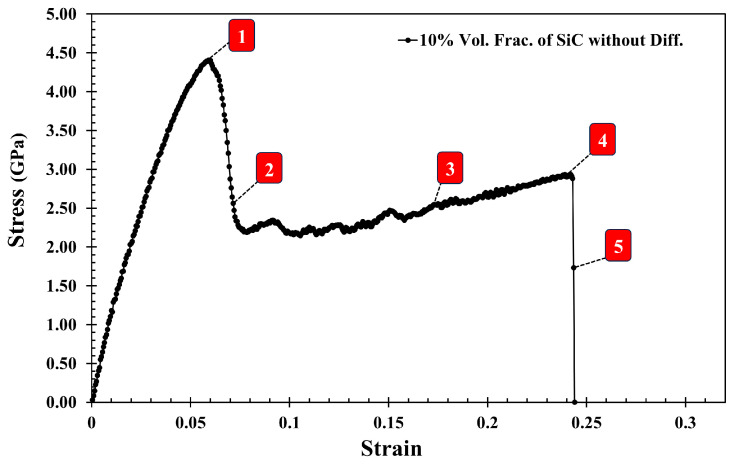
The tensile stress–strain curve of the Al-SiC composite with a 10% volume fraction of SiC in the z-direction $\left(\varepsilon_{zz}\right)$ without considering diffusion with marked characteristic points 1–5.

**Figure 7:**
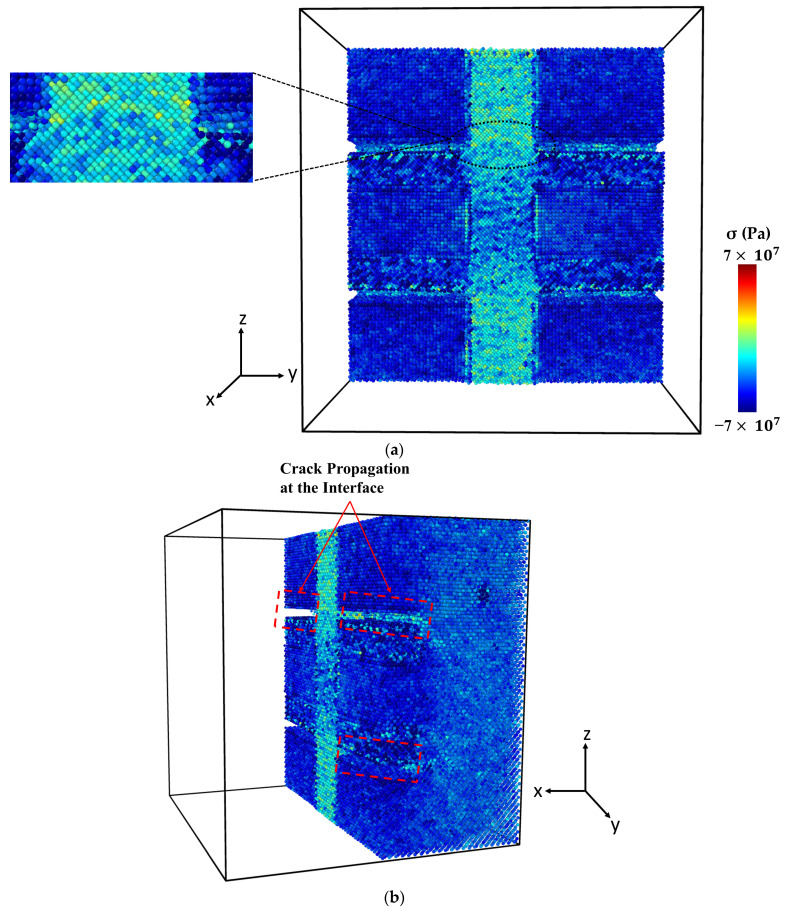
Point 1: The cross-section of an Al-SiC composite (10% volume fraction) at ε = 0.06 strain $\left(\varepsilon_{zz}\right)$ under uniaxial tensile loading ($\sigma_{zz})$ without considering diffusion: (**a**) cross-section without diffusion consideration, and (**b**) side view.

**Figure 8:**
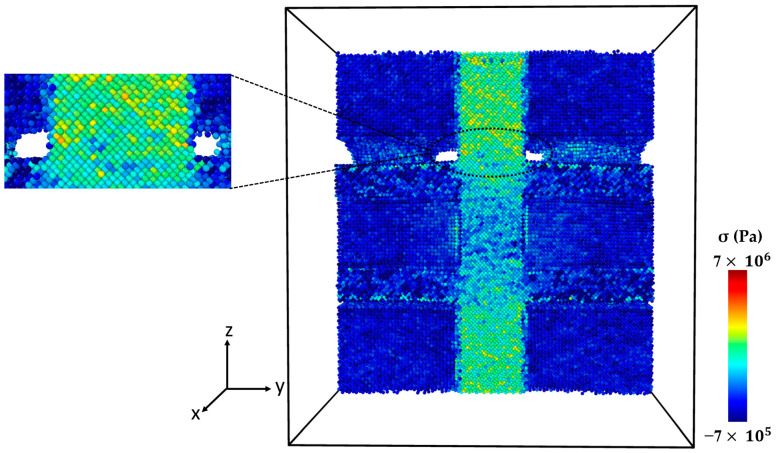
Point 2: The cross-section of an Al-SiC composite (10% volume fraction) at ε = 0.0755 strain $\left(\varepsilon_{zz}\right)$ under uniaxial tensile loading ($\sigma_{zz})$ without considering diffusion.

Figure 6 shows the stress–strain curve of the Al-SiC composite with a 10% volume fraction of SiC without considering diffusion. As can be observed in Figure 6, five points are highlighted on the curve. Point 1 is associated with the maximum strength of the composite, and Point 2 is marked with a significant reduction (jump). Point 3 is associated with the initiation of cracking, and Point 4 is related to initiating an initial crack on the SiC section. Point 5 is related to failure. Figure 7 illustrates a snapshot of the cross-section of the Al-SiC composite with a 10% volume fraction of SiC at Point 1, at ε = 0.06. Observing Figure 7, it is clear that debonding starts from the SiC sub-columns, and the main SiC column is not high stress. Therefore, fracture in this area begins at the interface between the SiC sub-columns and the Al matrix (Figure 7b). It could be due to the absence of diffusion, which results in a weak interface between SiC and Al, especially in the sub-columns. Point 2, marked in Figure 8, illustrates the growth of fractures at the interface of Al-SiC in the sub-columns, particularly on the top side of the simulation box.

**Figure 9:**
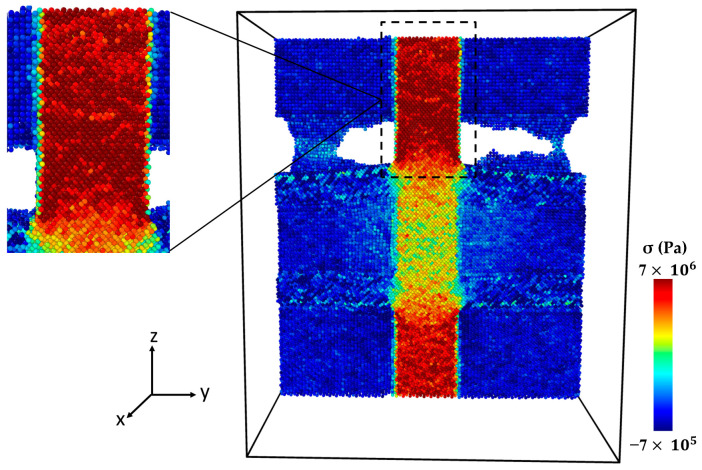
Point 3: The cross-section of an Al-SiC composite (10% volume fraction) at ε = 0.17 strain $\left(\varepsilon_{zz}\right)$ under uniaxial tensile loading without considering diffusion.

**Figure 10:**
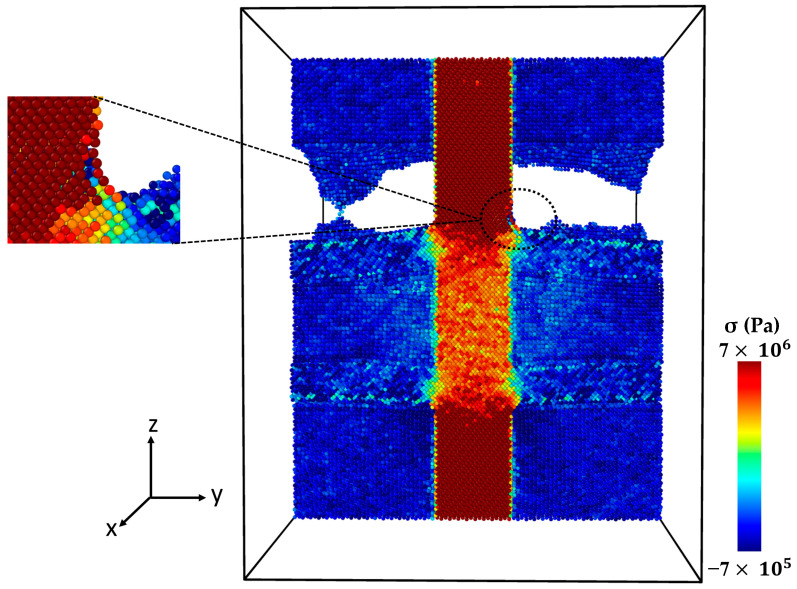
Point 4: The cross-section of an Al-SiC composite (10% volume fraction) at ε = 0.0243 strain $\left(\varepsilon_{zz}\right)$ under uniaxial tensile loading ($\sigma_{zz})$ without considering diffusion.

**Figure 11:**
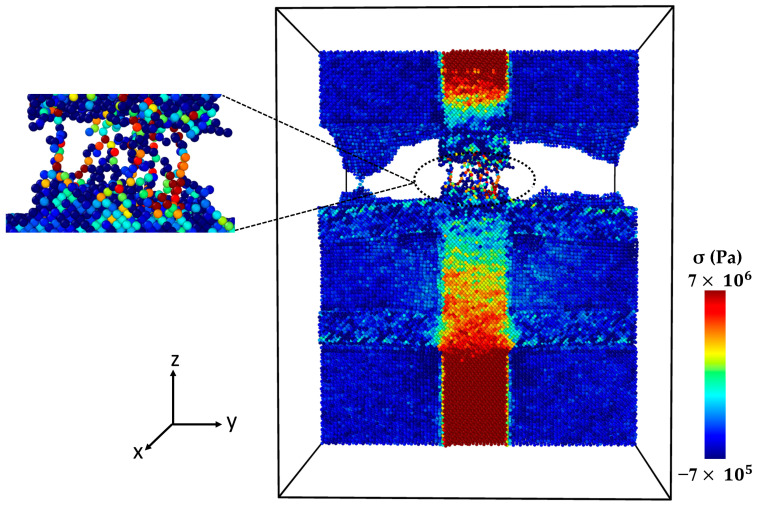
Point 5: The cross-section of an Al-SiC composite (10% volume fraction) at ε = 0.02435 strain $\left(\varepsilon_{zz}\right)$ under uniaxial tensile loading ($\sigma_{zz})$ without considering diffusion.

Necking under tensile loading occurs between Points 2 and 4, causing the composite to experience deformation with a narrowed cross-section (Point 3) at the main SiC column (Figure 9). The presence of debonding and defects, particularly at the interface between the Al matrix and SiC particles, contributed to the formation of these necked regions [28,29]. Figure 10 displays the initial crack on the main SiC column (Point 4). The maximum local stress (red area) has occurred at this point. The separation between the Al and SiC sub-columns has wholly occurred. The initial fracture on the main SiC column occurred at its corner. Figure 11 depicts the failure of the Al-SiC composite at Point 5. In Figure 11, the Al-SiC bonds are debonded not only in the sub-columns but also in the main SiC column.

**Figure 12:**
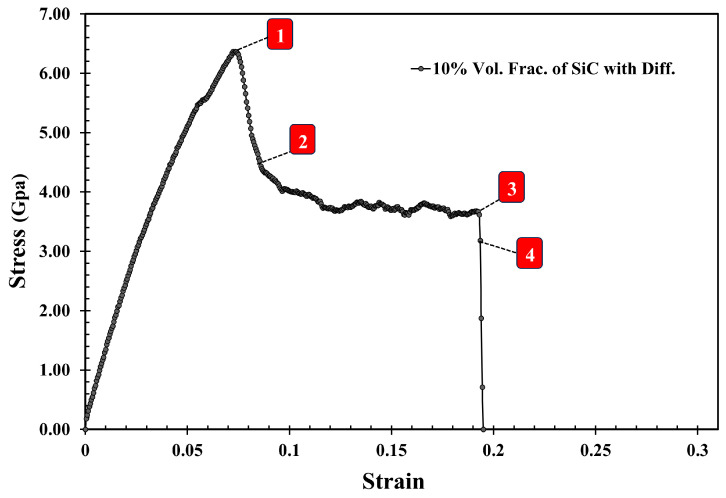
The tensile stress–strain curve of the Al-SiC composite with a 10% volume fraction of SiC in the z-direction $\left(\varepsilon_{zz}\right)$ considering diffusion with marked characteristic points 1–4.

**Figure 13:**
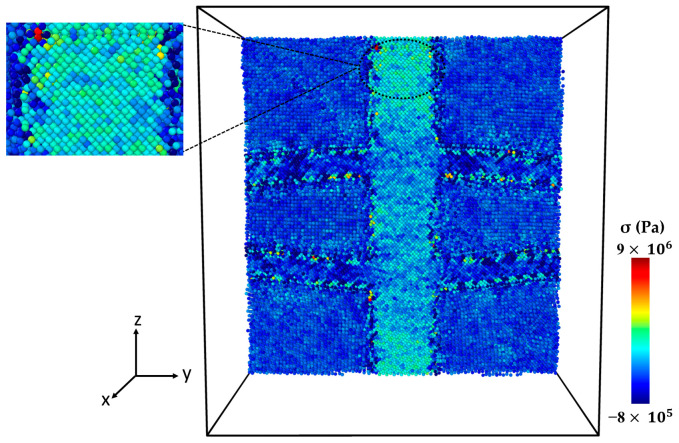
Point 1: The cross-section of an Al-SiC composite (10% volume fraction) at ε = 0.0735 strain $\left(\varepsilon_{zz}\right)$ under uniaxial tensile loading ($\sigma_{zz})$ considering diffusion.

**Figure 14:**
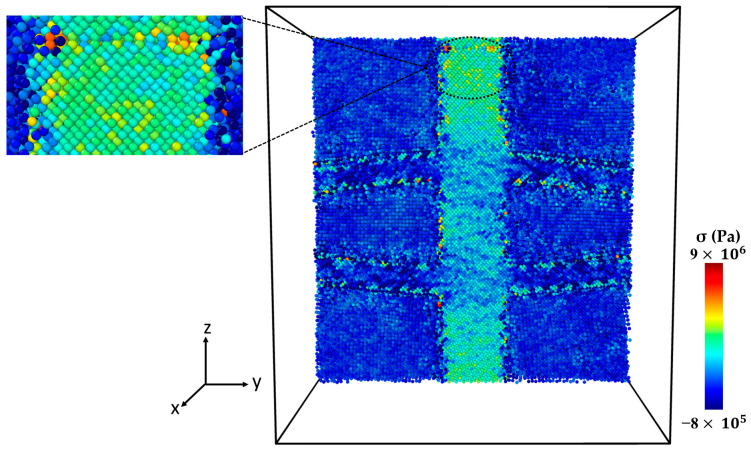
Point 2: The cross-section of an Al-SiC composite (10% volume fraction) at ε = 0.087 strain $\left(\varepsilon_{zz}\right)$ under uniaxial tensile loading ($\sigma_{zz})$ considering diffusion.

Figure 12 depicts the stress–strain curve of the Al-SiC composite with a 10% volume fraction of SiC in the z-direction, considering the effects of diffusion. Four points are marked in this diagram (Point 1, Point 2, Point 3, and Point 4). Figure 13 (Point 1) is a cross-section view of the Al-SiC composite with a 10% volume fraction of SiC, considering the diffusion process and showing the maximum strength. Unlike the non-diffusion case, in this section, the separation of Al and SiC at the interfaces is not observed either in the side columns or the main column. Figure 14 shows that with increasing strain at Point 2 (ε = 0.087), the local stress in the SiC main column increases. 

**Figure 15:**
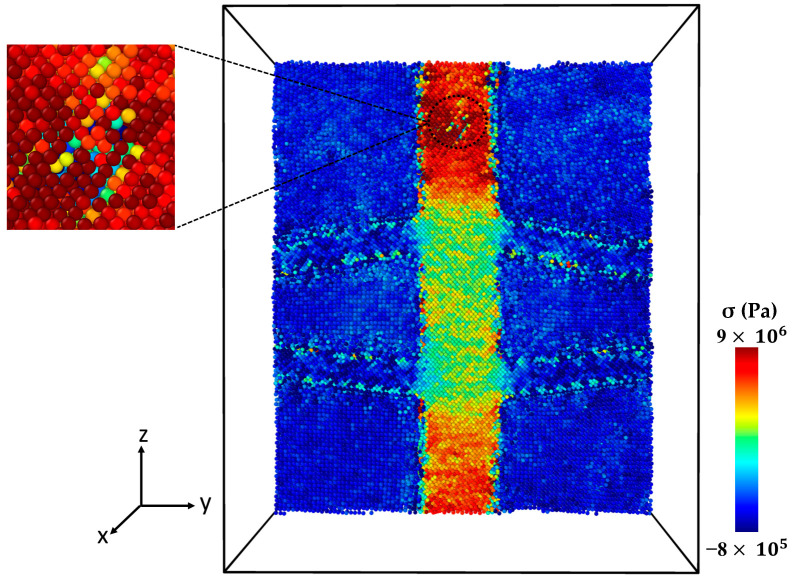
Point 3: The cross-section of an Al-SiC composite (10% volume fraction) at ε = 0.193 strain $\left(\varepsilon_{zz}\right)$ under uniaxial tensile loading ($\sigma_{zz})$ considering diffusion.

**Figure 16:**
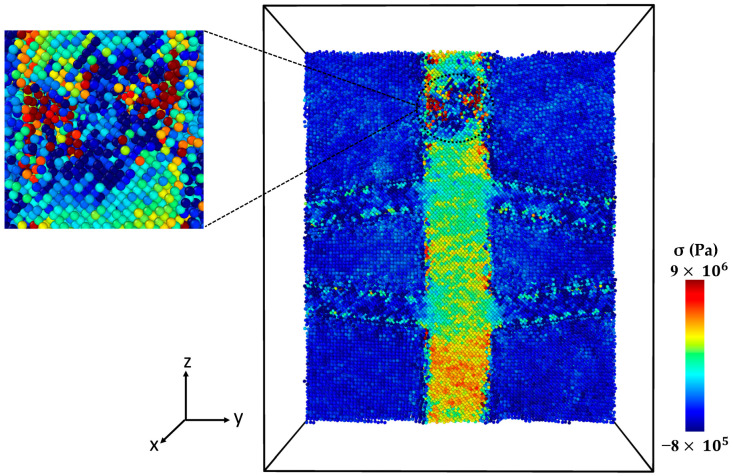
Point 4: The cross-section of an Al-SiC composite (10% volume fraction) at ε = 0.1935 strain $\left(\varepsilon_{zz}\right)$ under uniaxial tensile loading ($\sigma_{zz})$ considering diffusion.

This increasing local stress continues until it reaches Point 3 (Figure 15), which causes an initial crack in the main SiC column. As can be observed in Figure 16, eventually at Point 4, failure occurred. Referring to Figure 13, Figure 14, Figure 15 and Figure 16 and previous Figures (Figure 7, Figure 8, Figure 9, Figure 10 and Figure 11), it is evident that diffusion affects the interface between the Al atoms and SiC atoms, ultimately impacting the strength of the composite.

## 3. Methodology

As stated previously, this study aimed to develop a complex Al-SiC model to investigate the mechanical properties of the Al-SiC composite under uniaxial tensile loading, with and without considering diffusion, using MD simulation.

In this study, molecular dynamics simulations were conducted using the open-source program LAMMPS (Large-scale Atomic/Molecular Massively Parallel Simulator) [15], and the evolution of the atomic structure was visualized using the open visualization tool OVITO (3.10.5 version) [30]. In the MD method, atomic interactions are essential for calculating the kinematic parameters of particles according to Newton’s second law. Consequently, the atomistic model must include suitable potential functions representing these atomic interactions. The parameters of a potential function can be obtained from ab initio calculations or experimental data. In the following section, the interatomic potentials of Al and SiC as well as the interface between them will be explained.

### 3.1. Potential Functions

The Embedded Atom Method (EAM) potential is widely used in the simulations of metallic systems, allowing for the accurate exploration of various mechanical, thermal, electrical, and chemical properties. This study used the EAM potentials proposed by Mendelev et al. [31] and Mishin et al. [32] to model the interatomic forces between aluminum atoms.

The Tersoff potential [33] is widely employed in the literature to describe interactions between Si and C atoms. This potential is a bond-order potential, where the potential energy between atoms *i* and *j* is expressed as a function of the local atomic environment, including bond lengths and angles.

(3) $$E=\sum_{i}E_{i}=\frac{1}{2}\sum_{i\neq j}f_{c}\left(r_{ij}\right)\left[f_{R}\left(r_{ij}\right)+b_{ij}f_{A}\left(r_{ij}\right)\right]\;$$

where, $r_{ij}\;$ represents the bond length between atoms *i* and *j*, while $f_{R}\left(r_{ij}\right)$, $f\left(r_{ij}\right)$, and $f_{A}\left(r_{ij}\right)$ represent the repulsive and attractive atom pair interactions and the optional cutoff function that determines the interaction range, respectively. Additionally, $b_{ij}$ is a function that adjusts the attractive interaction and includes many-body interactions.

The Morse potential was employed to model the Al-SiC interface, representing a two-body pairwise potential. The Morse potential is expressed as follows:

(4) $$V=D_{0}\left[e^{-2\alpha (r-r_{0})}-2e^{-\alpha (r-r_{0})}\right]\;$$

where $D_{0},\;r_{0}$, r, and *α* represent the distance between atoms, the equilibrium bond length, the well depth of the potential, and the width of the potential, respectively. The references [27,34,35] provide the parameters of the Morse potential for Al–C and Al-Si interactions. The parameters used in the current simulations are given in Table 4.

### 3.2. Molecular Dynamics Model

**Figure 17:**
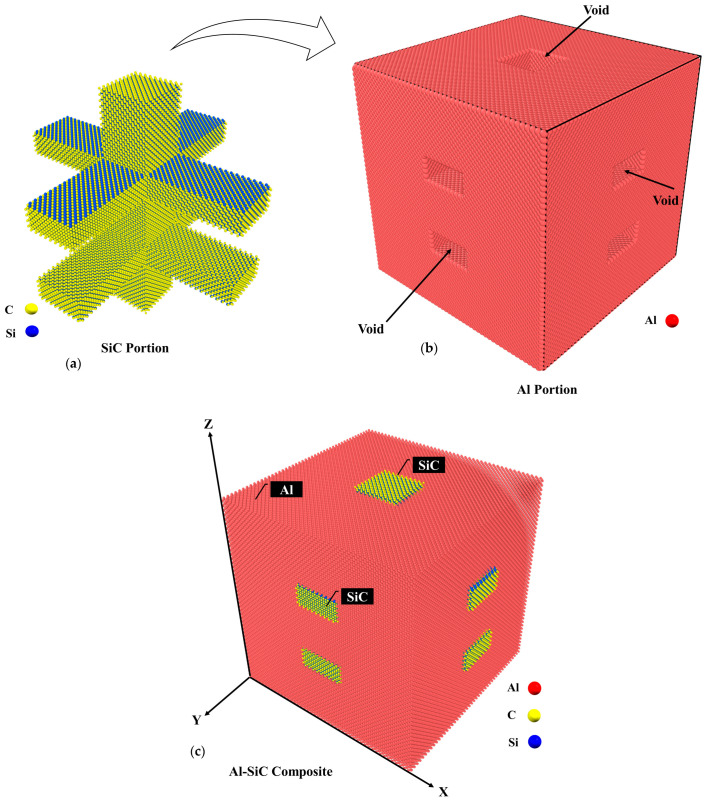
Images of (**a**) the SiC portion, (**b**) the Al portion containing void spaces, and (**c**) the Al-SiC composite after the merging process.

In this study, the cubic (3C-SiC) form of SiC crystal was used to generate the SiC portion of the models. Initially, the SiC models were generated separately, and voids were created in the aluminum simulation box based on SiC dimensions. Subsequently, the SiC structure was merged with pure aluminum (see Figure 17).

Selecting the appropriate dimensions for the MD simulation box became a crucial factor for achieving reasonable accuracy and computational efficiency. The MD model was configured with the dimensions of approximately 186.3 × 186.3 × 186.3 Å in the x, y, and z axes. The number of atoms in the Al-SiC simulation boxes is shown in Table 5. Periodic boundary conditions were applied in the x- and y-directions, while non-periodic boundary conditions were used in the z-direction to eliminate edge effects.

As mentioned, this study aimed to investigate the mechanical properties of a complex model of an Al-SiC composite under uniaxial tensile loading, with and without considering diffusion. For this purpose, two different scenarios were employed: first, uniaxial tensile testing at room temperature (300 K), and second, uniaxial tensile testing with the presence of diffusion. For the diffusion process, the system is initially heated to a temperature of 1500 K and maintained at this temperature for a duration of 1.0 ns, then cooled back to room temperature.

The geometric configuration of all the samples was initially optimized using the conjugate gradient energy minimization algorithm with a specified energy tolerance of 1 × $10^{-10}$ and a force tolerance of 1 × $10^{-10}$ eV/Å. The NVT ensemble was then applied to the sample at a constant temperature of 300 K for 40 ps, allowing for the volume adjustment and relaxation of the assembled interface system.

After energy minimization and relaxation, the sample was subjected to an NVT ensemble at a constant temperature of 300 K for 50 ps to equilibrate the system. Subsequently, the system was prepared for the uniaxial tensile deformation process.

To simulate the heat treatment behavior of the systems, an isothermal–isobaric (NPT) ensemble was employed at a constant pressure of zero (0 Pa) and a temperature of 300 K for a duration of 40 ps. This equilibration step allowed the volume to adjust and the assembled interface system to relax. Additionally, the sample underwent gradual heating at a 1 K/ps rate, reaching a final temperature of 1500 K. After that, the temperature was kept at 1500 K for 1.0 ns. As a final step, the samples were cooled down to 300 K at a rate of 1 K/ps. Then, a structural relaxation process was conducted for 30 ps under zero pressure and 300 K to eliminate internal residual stresses. The NPT ensemble with zero pressure was considered for all of these processes. The system was to apply uniaxial tensile loading.

### 3.3. Loading Conditions

The simulation box was subjected to tensile loading following equilibration to generate stress–strain curves. All the simulations were conducted at a strain rate of 5 × 10^8^ S^−1^. A simulation time step of 1 fs was employed to ensure highly accurate results.

## 4. Conclusions

The impact of diffusion on the mechanical properties of a complex Al-SiC composite under a uniaxial tensile test was investigated via MD simulation. Three different volume fractions of SiC structure were examined, including 10%, 15%, and 25% SiC. Tensile tests were conducted in two directions: along the x-axis and the z-axis. The entire test was conducted once in the presence of diffusion and once in the absence of diffusion. Based on the present computations, the following conclusions can be drawn:

- As the volume fraction of SiC increased, Young’s modulus of the Al-SiC composite in all the models showed a significant increase.
- The Al-SiC composite strength rose dramatically with increasing SiC volume fraction in all the models.
- The results indicate that diffusion does not significantly affect Young’s modulus of the Al-SiC composite when compared to the scenarios without considering diffusion. However, diffusion does result in a notable increase in the strength of the composite.
- The strength properties of the Al-SiC composite were better along the z-axis than the x-axis, potentially due to its unique geometric shape.

Overall, the results demonstrated that diffusion can promote strong bonding in the interface area, directly influencing the mechanical behavior of the composite.

## Figures and Tables

**Table 1:** The Young’s modulus of Al-SiC at the different volume fractions of SiC with and without diffusion.

	Volume Fraction of SiC Phase	Young’s Modulus (GPa)
	10%	123.72
With diffusion	15%	135.21
	25%	141.32
	10%	120.42
Without diffusion	15%	132.32
	25%	139.11

**Table 2:** The tensile ultimate strength and toughness of Al-SiC at different volume fractions of SiC with and without diffusion in the z-direction.

	Volume Fraction of SiC Phase	Tensile Ultimate Strength (GPa)	Toughness (GPa)
With diffusion	10%	5.93	0.863
	15%	7.67	1.296
	25%	11.29	2.086
Without diffusion	10%	4.105	0.628
	15%	6.110	1.206
	25%	11.28	1.947

**Table 3:** The tensile ultimate strength and toughness of Al-SiC at different volume fractions of SiC with and without diffusion in the x-direction.

	Volume Fraction of SiC Phase	Tensile Ultimate Strength (GPa)	Toughness (GPa)
	10%	5.98	0.759
With diffusion	15%	6.35	0.768
	25%	6.63	0.557
	10%	5.04	0.857
Without diffusion	15%	5.12	0.673
	25%	5.31	0.788

**Table 4:** Parameterized Morse potential function parameters derived from references [27,34,35].

System	Parameters	Value
Al-SiC	$D_{0}$ (eV)	0.4824
	*α* (1/Å)	1.322
	$r_{0}$ (1/Å)	2.92
Al-C	$D_{0}$ (eV)	0.4691
	*α* (1/Å)	1.738
	$r_{0}$ (1/Å)	2.246

**Table 5:** Number of atoms for Al-SiC composite with three volume fractions (10%, 15%, and 25%) of SiC.

Volume Fraction (%)	Type of Atom	Number of Atoms
10%	Al	342,144
	Si	35,964
	C	38,960
15%	Al	325,458
	Si	52,504
	C	51,527
25%	Al	298,178
	Si	71,928
	C	76,648

## Data Availability

Data is available on request.
